# A Simple Algorithm for Assimilating Marker-Based Motion Capture Data During Periodic Human Movement Into Models of Multi-Rigid-Body Systems

**DOI:** 10.3389/fbioe.2018.00141

**Published:** 2018-10-18

**Authors:** Yasuyuki Suzuki, Takuya Inoue, Taishin Nomura

**Affiliations:** Division of Mechanical Science and Bioengineering, Graduate School of Engineering Science, Osaka University, Osaka, Japan

**Keywords:** motion analysis, soft tissue artifact, error reduction, stereophotogrammetry, data assimilation

## Abstract

Human movement analysis is often performed with a model of multi-rigid-body system, whereby reflective-marker-based motion capture data are assimilated into the model for characterizing kinematics and kinetics of the movements quantitatively. Accuracy of such analysis is limited, due to motions of the markers on the skin relative to the underlying skeletal system, referred to as the soft tissue artifact (STA). Here we propose a simple algorithm for assimilating motion capture data during periodic human movements, such as bipedal walking, into models of multi-rigid-body systems in a way that the assimilated motions are not affected by STA. The proposed algorithm assumes that STA time-profiles during periodic movements are also periodic. We then express unknown STA profiles using Fourier series, and show that the Fourier coefficients can be determined optimally based solely on the periodicity assumption for the STA and kinematic constraints requiring that any two adjacent rigid-links are connected by a rotary joint, leading to the STA-free assimilated motion that is consistent with the multi-rigid-link model. To assess the efficiency of the algorithm, we performed a numerical experiment using a dynamic model of human gait composed of seven rigid links, on which we placed STA-affected markers, and showed that the algorithm can estimate the STA accurately and retrieve the non-STA-affected true motion of the model. We also confirmed that our STA-removal processing improves accuracy of the inverse dynamics analysis, suggesting the usability of the proposed algorithm for gait analysis.

## 1. Introduction

Human movement analysis is performed in various fields, including biomechanics, physiology, orthopedics, neurology, and sports science, playing an important role for understanding physical functions, motor control, and motor dysfunctions (e.g., see Harris and Smith, 1996; Winter, 2009; Lu and Chang, 2012 for a review). Optoelectronic stereo-photogrammetry, referred simply to as the motion capture system, is the most popular method used in the human movement analysis, in which spatio-temporal changes in positions of reflective markers placed on anatomical landmarks of the human body, allow us to describe the body movement quantitatively in the computer. In this process, the human body is often modeled by a multi-link-rigid-body system, and reflective-marker-based motion capture data are assimilated into the model for characterizing kinematics and kinetics of the movements quantitatively as well as mechano-dynamic analysis of the movement such as the inverse dynamics analysis to estimate joint torque profiles along the movement (Sibella et al., 2003; Ren et al., 2008; Alexander and Schwameder, 2016).

A goal of the data assimilation process is to make the motions captured from an experimental subject physically consistent with those of the model, while they are as close as to the actual motions of the subject (Cappozzo et al., 2005). This is because, if no assimilation processing is performed, the original, non-assimilated motion data exhibit several inconsistencies with the model, such as temporal variations in the length of each link, which should not happen under the rigid-body-model assumption. The underlying cause lowering an accuracy of the assimilation is the fact that the human body is not a simple multi-rigid-body system: skeletal limbs are covered by deformable muscular-tissues, the trunk, foot, etc., are composed of a number of small bones despite a common modeling simplification with single or a few links, and no joints are rotary nor spherical. Nevertheless, the multi-rigid-body modeling is still useful for the quantitative human movement analysis (Dicharry, 2010; Rusaw and Ramstrand, 2011). Moreover, for practical applications, it is preferable that the accurate data assimilation can be performed with a small number of markers used for the motion capture (Simon, 2004).

In this study, we consider the minimum numbers of markers necessary for identifying a position and a posture of each link, and movements of the markers on the skin relative to the underlying skeletal system, referred to as the soft tissue artifact (STA), as one of the major causes that lower accuracy of the assimilation (Camomilla et al., 2017). STA has its origin in wobbling of the soft tissue, stretching of the skin, and activation of the muscles. STA-characteristics have been investigated by comparing movements of markers attached on the skin with those of markers attached to intra-cortical pins fixed directly into the bones (Lafortune et al., 1992; Benoit et al., 2006; Andersen et al., 2012; Blache et al., 2017), to a splint fixed to the bone (Cappozzo et al., 1996), and to the Percutaneous Skeletal Tracker (Holden et al., 1997). Trials for measuring movements of the bone using X-ray (Sati et al., 1996; Li et al., 2017) and MRI have been also carried out (Ryu et al., 2006). Moreover, a number of computational methodologies have been proposed to minimize and/or compensate the effects of STA, which include the multiple anatomical landmark calibration (Cappello et al., 1997, 2005), the dynamic calibration (Lucchetti et al., 1998), the point cluster technique (Andriacchi et al., 1998; Alexander and Andriacchi, 2001), the multi body optimization (Lu and O'Connor, 1999; Yoshikawa et al., 2012, 2013; Clément et al., 2015; Richard et al., 2017), approaches with Kalman filters (Cerveri et al., 2003, 2005; Halvorsen et al., 2008; Bonnet et al., 2017b), an optimization using the ground reaction forces (Riemer et al., 2008), the linear 3D interpolation and approximation methods (Dumas and Cheze, 2009), among others. However, reliable yet simple assimilations of skeletal motions have not been achieved satisfactorily (Leardini et al., 2005; Peters et al., 2010; Camomilla et al., 2017).

Primary causes of the difficulty to minimize and/or compensate the effects of STA are associated with the fact that the patterns of the artifacts are task-dependent, although the STA is reproducible within a given specific task, but not among subjects (Leardini et al., 2005). Since most of the methods proposed in literatures so far need to be task-adjusted, they require a precise calibration and tuning in numerical compensations specifically for each subject and each task. Thus, it would be very useful if an algorithm focuses only on the relationship between STA and skeletal motions in common across movements, independent of tasks.

Previously, we studied motions of markers attached on the anatomical landmarks during human walking, and showed that STA profiles were also periodic (Inoue et al., 2016). In this paper, we propose a novel and simple algorithm to assimilate motion-captured periodic human movements, represented by positions of markers, that are affected by STA into models of multiple rigid-link systems. In section 2, we describe the proposed algorithm using a simple example of a two-link model that moves in the two-dimensional space. We then performed a numerical experiment using a model of human walking to assess the efficiency of the algorithm, for which methods for the simulation are summarized in section 3. Section 4 reports the results of the numerical experiment. This is a preliminary study that aims to develop a theoretical foundation of the algorithm in three-dimensional system. We discuss whether the algorithm can be applied to posture estimation in three-dimensional space in section 5.

## 2. The proposed algorithm

### 2.1. Problem setting

**Figure 1:**
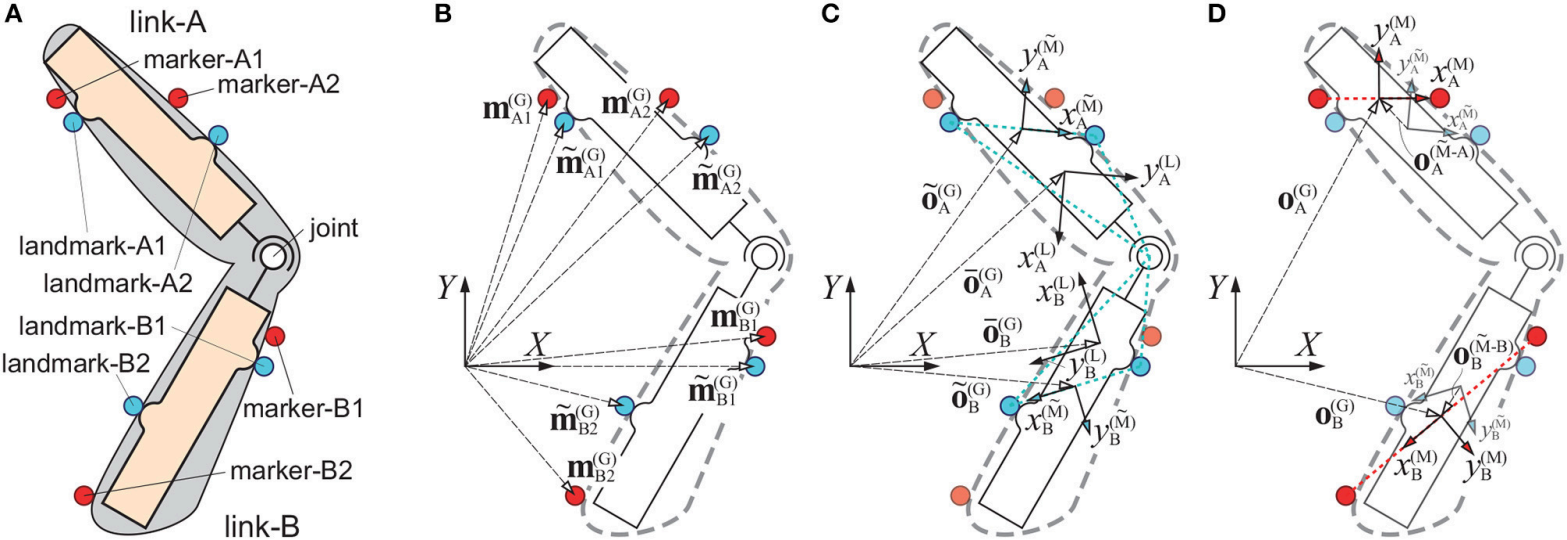
Two-link model, which is used to explain posture estimation algorithm. The two-link model consists of two rigid links, and surrounded by soft tissue. In each panel, blue circles represent markers located exactly on the landmarks of the two-link model, and red circles represent markers captured by motion capture system, which excurse due to STA. **(A)** Conceptual diagram of two-link model. Brown rectangular areas are rigid links, and gray shaded region is soft tissue. **(B)** Position vectors of landmark markers [${\tilde{\text{m}}}_{ij}^{\left(\text{G}\right)}$, *i* = {A, B}, *j* = {1, 2}], and captured markers [${\text{m}}_{ij}^{\left(\text{G}\right)}$]. **(C)** Position vectors of local coordinate system [the link coordinate (${\bar{\text{o}}}_{i}^{\left(\text{G}\right)}$) and the landmark coordinate (${\tilde{\text{o}}}_{i}^{\left(\text{G}\right)}$)] and coordinate axes of those in global coordinate system [$x_{i}^{\left(\text{L}\right)}$-$y_{i}^{\left(\text{L}\right)}$ and $x_{i}^{\left(\tilde{\text{M}}\right)}$-$y_{i}^{\left(\tilde{\text{M}}\right)}$]. **(D)** Position vectors of the marker coordinate system in global coordinate system [${\text{o}}_{i}^{\left(\text{G}\right)}$] and in the landmark coordinate system [${\text{o}}_{i}^{\left(\tilde{\text{M}}-i\right)}$], and coordinate axes of that [$x_{i}^{\left(\text{M}\right)}$-$y_{i}^{\left(\text{M}\right)}$].

In this section, we consider temporal changes in a posture of a planar two-link system that moves periodically. This is just for simplicity, and the algorithm proposed here can be applied to systems with more links as shown later in this paper, and could extended for three-dimensional systems. The two-link model consists of two rigid links, referred to as the link-A and the link-B as shown in Figure 1A, which are connected to each other by a pin-joint. We consider a situation such that a soft tissue (the gray area in Figure 1A) surrounds the rigid-link system. The minimum number of markers necessary for specifying the position and posture of each link is two for the planar system. Thus two markers, referred to as the marker-*i*1 and the marker-*i*2 for *i*={A,B}, are attached on the surface of the soft tissue. We assume that those markers are attached accurately to the landmarks of each link (the landmarks-A1 and A2 for the link-A and the landmarks-B1 and B2 for the link-B) when the system is at rest without any movement. However, each marker may excurse from the landmark, causing STA during periodic movements of the system.

We denote by ${\text{m}}_{ij}^{\left(\text{G}\right)}\left[n\right]$ the spatio-temporal position of the marker-*ij* in the global coordinate system, where the superscript (G) represents the global coordinate *X*-*Y* as in Figure 1B for *i*={A,B} and *j*={1,2}. The integers *n* = 1, 2, ⋯  indicate the data numbers or the sampling times. ${\text{m}}_{ij}^{\left(\text{G}\right)}\left[n\right]$ corresponds to an experimentally motion-captured positions of the marker-*ij* contaminated by unknown STA. Now, the positions of the markers located exactly on the landmarks are denoted as ${\tilde{\text{m}}}_{ij}^{\left(\text{G}\right)}\left[n\right]$ (Figure 1B), which cannot be measured experimentally, but can be estimated if we can estimate the unknown STA time-profiles as ${\tilde{\text{m}}}_{ij}^{\left(\text{G}\right)}\left[n\right]={\text{m}}_{ij}^{\left(\text{G}\right)}\left[n\right]-\text{STA}$. The problem here can be stated as follows: the motion capture experiments provide time-series data of ${\text{m}}_{ij}^{\left(\text{G}\right)}\left[n\right]$, from which we would like to estimate the positions and postures of the link-A and the link-B at each time instant so that they are consistent with the assumption for the two-rigid-link model. This may be possible if we can estimate the STA profiles and then ${\tilde{\text{m}}}_{ij}^{\left(\text{G}\right)}\left[n\right]$, as described in this sequel.

### 2.2. Definitions of marker-positions and the multi-link system

We define a local coordinate system, referred to as *the link-coordinate system*, which is fixed on the link-*i* as in Figure 1C to describe the position and posture of the link-*i*. The link-coordinate system of the link-*i* is denoted by $x_{i}^{\left(\text{L}\right)}$-$y_{i}^{\left(\text{L}\right)}$, where the superscript (L) represents the link-coordinate system. Position of the origin and the posture of the link-coordinate changes in the global coordinate system together with translation and rotation of the link-*i*. The origin of the link-coordinate system ${\bar{\text{o}}}_{i}^{\left(\text{G}\right)}\left[n\right]$ represents the global position of the link-*i*, and the coordinate transformation matrix ${\bar{\text{A}}}_{i}^{\left(\text{G}\right)}\left[n\right]$, consisting of normalized orthogonal basis vectors in the directions of $x_{i}^{\left(\text{L}\right)}$- and $y_{i}^{\left(\text{L}\right)}$-axes, represents the posture of the link-*i*. We set the link-coordinate system of the link-*i* such that the origin is located at the centroid of the triangle having the vertices at the joint and two landmarks (${\tilde{\text{m}}}_{i1}^{\left(\text{G}\right)}\left[n\right]$ and ${\tilde{\text{m}}}_{i2}^{\left(\text{G}\right)}\left[n\right]$). As in Figure 1C, the $y_{i}^{\left(\text{L}\right)}$-axis of the link-coordinate system of the link-*i* is defined so that it is parallel to the vector directing from ${\tilde{\text{m}}}_{i1}^{\left(\text{G}\right)}\left[n\right]$ to ${\tilde{\text{m}}}_{i2}^{\left(\text{G}\right)}\left[n\right]$, and then the $x_{i}^{\left(\text{L}\right)}$-axis is defined so that it becomes orthogonal to the $y_{i}^{\left(\text{L}\right)}$-axis^1^. It is essential for the proposed algorithm to notice that ${\bar{\text{o}}}_{i}^{\left(\text{G}\right)}\left[n\right]$ and ${\bar{\text{A}}}_{i}^{\left(\text{G}\right)}\left[n\right]$ of the link-coordinate system defined here cannot be determined easily from the motion-captured marker data ${\text{m}}_{ij}^{\left(\text{G}\right)}\left[n\right]$, since three vertices at the joint, ${\tilde{\text{m}}}_{i1}^{\left(\text{G}\right)}\left[n\right]$ and ${\tilde{\text{m}}}_{i2}^{\left(\text{G}\right)}\left[n\right]$ are all unknown. That is, the maker positions ${\text{m}}_{ij}^{\left(\text{G}\right)}\left[n\right]$ may show temporal deviations from the landmark positions ${\tilde{\text{m}}}_{ij}^{\left(\text{G}\right)}\left[n\right]$ due to unknown STA, and thus the landmark positions ${\tilde{\text{m}}}_{i1}^{\left(\text{G}\right)}\left[n\right]$ and ${\tilde{\text{m}}}_{i2}^{\left(\text{G}\right)}\left[n\right]$ are not available directly from the marker positions. Obviously, the joint position needs to be estimated somehow from the STA-affected marker positions.

If all markers were strictly fixed on the landmarks of each link, i.e., if the motion-captured marker positions were not affected by STA, the link-coordinate systems could be estimated easily, since the landmark-marker positions ${\tilde{\text{m}}}_{ij}^{\left(\text{G}\right)}\left[n\right]$ are exactly the same as the motion-captured marker positions ${\text{m}}_{ij}^{\left(\text{G}\right)}\left[n\right]$, and the position of the joint could be calculated as described below.

#### 2.2.1. Estimation of the joint position from Non-STA-affected markers

For estimating the joint position in the global coordinate system, we define another local coordinate system based on the landmark positions ${\tilde{\text{m}}}_{ij}^{\left(\text{G}\right)}\left[n\right]$ (Figure 1C), referred to as *the landmark-coordinate system* and denoted by $x_{i}^{\left(\tilde{\text{M}}\right)}$-$y_{i}^{\left(\tilde{\text{M}}\right)}$. The origin of the landmark-coordinate system in the global coordinate system, ${\tilde{\text{o}}}_{i}^{\left(\text{G}\right)}\left[n\right]$ for the link-*i*, is set as the midpoint of the landmark-*i*1 and the landmark-*i*2, and the $x_{i}^{\left(\tilde{\text{M}}\right)}$-axis of the landmark-coordinate system relative to the global coordinate system, which determines the posture matrix ${\tilde{\text{A}}}_{i}^{\left(\text{G}\right)}\left[n\right]$, is set as the normalized vector directing from the landmark-*i*1 to the landmark-*i*2:

(1) $$\begin{array}{rcl} {\tilde{\text{o}}}_{i}^{\left(\text{G}\right)}\left[n\right] & = & \frac{{\tilde{\text{m}}}_{i1}^{\left(\text{G}\right)}\left[n\right]+{\tilde{\text{m}}}_{i2}^{\left(\text{G}\right)}\left[n\right]}{2}, \end{array}$$

(2) $${\tilde{A}}_{i}^{\left(\text{G}\right)}[n]=\frac{1}{\left|{\tilde{m}}_{i\text{-diff}}^{\left(\text{G}\right)}[n]\right|}\left[\begin{array}{cc} {\tilde{m}}_{i\text{-diff}}^{\left(\text{G}\right)}[n] & \overset{|}{\underset{|}{|}}\;R_{\frac{\pi }{2}}{\tilde{m}}_{i\text{-diff}}^{\left(\text{G}\right)}[n] \end{array}\right],$$

where

$$\begin{array}{l} {\tilde{m}}_{i\text{-diff}}^{\left(\text{G}\right)}[n]={\tilde{m}}_{i2}^{\left(\text{G}\right)}[n]-{\tilde{m}}_{i1}^{\left(\text{G}\right)}[n],\\ \;R_{\frac{\pi }{2}}=\left[\begin{array}{cc} 0 & -1\\ 1 & 0 \end{array}\right]. \end{array}$$

The joint position in the landmark-coordinate system of the link-*i* is constant in time during any movement of the model, because the skeletal part of the link-*i* (each of the brown rectangular areas in Figure 1A) is assumed to be rigid body and the joint is pinning such two rigid bodies. Thus, we denote it by a time-independent constant vector ${\tilde{\text{j}}}_{i}^{\left(\tilde{\text{M}}\text{-}i\right)}$, where the superscript ($\tilde{\text{M}}$-*i*) means that the vector is represented in the landmark-coordinate system for the link-*i*. The joint position in the global coordinate system, denoted as the time-varying vector ${\tilde{\text{j}}}_{i}^{\left(\text{G}\right)}\left[n\right]$, can be described by the position and the posture of the landmark-coordinate system of the link-*i* as follows:

(3) $$\begin{array}{r} {\tilde{\text{j}}}_{i}^{\left(\text{G}\right)}\left[n\right]={\tilde{\text{o}}}_{i}^{\left(\text{G}\right)}\left[n\right]+{\tilde{\text{A}}}_{i}^{\left(\text{G}\right)}\left[n\right]{\tilde{\text{j}}}_{i}^{\left(\tilde{\text{M}}\text{-}i\right)}. \end{array}$$

Since the link-A and the link-B are connected by the joint at a single point in the global coordinate system, referred to as the ${\tilde{\text{j}}}^{\left(\text{G}\right)}\left[n\right]$, the joint position described in the link-A and that in the link-B by Equation (3) must be equal to each other for any time instant, i.e.,

(4) $$\begin{array}{r} {\tilde{\text{j}}}^{\left(\text{G}\right)}\left[n\right]={\tilde{\text{o}}}_{\text{A}}^{\left(\text{G}\right)}\left[n\right]+{\tilde{\text{A}}}_{\text{A}}^{\left(\text{G}\right)}\left[n\right]{\tilde{\text{j}}}_{\text{A}}^{\left(\tilde{\text{M}}\text{-A}\right)}={\tilde{\text{o}}}_{\text{B}}^{\left(\text{G}\right)}\left[n\right]+{\tilde{\text{A}}}_{\text{B}}^{\left(\text{G}\right)}\left[n\right]{\tilde{\text{j}}}_{\text{B}}^{\left(\tilde{\text{M}}\text{-B}\right)}. \end{array}$$

This is *the joint constraint* that assures the two adjacent links are connected at a single joint. The two unknown constant vectors ${\tilde{\text{j}}}_{\text{A}}^{\left(\tilde{\text{M}}\text{-A}\right)}$ and ${\tilde{\text{j}}}_{\text{B}}^{\left(\tilde{\text{M}}\text{-B}\right)}$, representing the joint position in the landmark-coordinate system of the link-A and the link-B, can be obtained by solving Equation (4) at any two different time instances *n*1 and *n*2 as

(5) $$\left[\begin{array}{c} {\tilde{j}}_{\text{A}}^{\left(\tilde{\text{M}}\text{-A}\right)}\\ {\tilde{j}}_{\text{B}}^{\left(\tilde{\text{M}}\text{-B}\right)} \end{array}\right]={\left[\begin{array}{cc} {\tilde{A}}_{\text{A}}^{\left(\text{G}\right)}[n1] & -{\tilde{A}}_{\text{B}}^{\left(\text{G}\right)}[n1]\\ {\tilde{A}}_{\text{A}}^{\left(\text{G}\right)}[n2] & -{\tilde{A}}_{\text{B}}^{\left(\text{G}\right)}[n2] \end{array}\right]}^{-1}\left[\begin{array}{c} {\tilde{o}}_{\text{B}}^{\left(\text{G}\right)}[n1]-{\tilde{o}}_{\text{A}}^{\left(\text{G}\right)}[n1]\\ {\tilde{o}}_{\text{B}}^{\left(\text{G}\right)}[n2]-{\tilde{o}}_{\text{A}}^{\left(\text{G}\right)}[n2] \end{array}\right].$$

Then the joint position in the global coordinate system, ${\tilde{\text{j}}}^{\left(\text{G}\right)}\left[n\right]$, can be obtained by Equation (3). In this way, using the joint position ${\tilde{\text{j}}}^{\left(\text{G}\right)}\left[n\right]$ in the global coordinate system and the positions of two landmarks for each link ${\tilde{\text{m}}}_{i1}^{\left(\text{G}\right)}\left[n\right]$ and ${\tilde{\text{m}}}_{i2}^{\left(\text{G}\right)}\left[n\right]$ that are experimentally available in the special case with ${\tilde{\text{m}}}_{ij}^{\left(\text{G}\right)}\left[n\right]={\text{m}}_{ij}^{\left(\text{G}\right)}\left[n\right]$, we can determine the position ${\tilde{\text{o}}}_{i}^{\left(\text{G}\right)}\left[n\right]$ and the posture ${\tilde{\text{A}}}_{i}^{\left(\text{G}\right)}\left[n\right]$ of the link-*i*, completing the assimilation almost directly.

However, in practice, unlike the case without STA considered here, the motion-captured marker positions are not the same as those of the landmark positions.

### 2.3. A naive algorithm for simply minimizing the joint-constraint-errors

Previously, we studied a preliminary algorithm to estimate skeletal movement of human body from captured positions of markers, which are attached to anatomical landmarks (Yoshikawa et al., 2012, 2013), referred to as the naive algorithm. Performances of the naive algorithm would be compared with those of the new algorithm proposed in this paper. The naive algorithm could reduce the influences of STA effectively through a multi-body optimization process. However, the obtained solutions (the estimated motions) do not necessarily coincide with the actual motions of the multi-link system. In this paper, we propose a novel but still simple algorithm to better assimilate the motion-captured data into models of multi-rigid-links during human periodic motion by estimating the unknown STA profiles.

The naive algorithm does not utilize a notion of the landmarks and the landmark-coordinate systems, and the local coordinate systems are defined based on the positions of the marker-*i*1 and the marker-*i*2 in a similar way to the landmark-coordinate system by removing the tilde-signs from the equations, because the tilde-signs are reserved for the landmark-markers. We refer to this “pseudo” local coordinate system as *the marker-coordinate system*, where the origin and the posture of the coordinate system (${\text{o}}_{i}^{\left(\text{G}\right)}\left[n\right]$ and ${\text{A}}_{i}^{\left(\text{G}\right)}\left[n\right]$) are defined as follows.

(6) $$\begin{array}{rcl} {\text{o}}_{i}^{\left(\text{G}\right)}\left[n\right] & = & \frac{{\text{m}}_{i1}^{\left(\text{G}\right)}\left[n\right]+{\text{m}}_{i2}^{\left(\text{G}\right)}\left[n\right]}{2}, \end{array}$$

(7) $$A_{i}^{\left(\text{G}\right)}[n]=\frac{1}{\left|m_{i\text{-diff}}^{\left(\text{G}\right)}[n]\right|}\left[\begin{array}{cc} m_{i\text{-diff}}^{\left(\text{G}\right)}[n] & \overset{|}{\underset{|}{|}}\;R_{\frac{\pi }{2}}m_{i\text{-diff}}^{\left(\text{G}\right)}[n] \end{array}\right],$$

where

$$\begin{array}{l} {\text{m}}_{i\text{-diff}}^{\left(\text{G}\right)}\left[n\right]={\text{m}}_{i2}^{\left(\text{G}\right)}\left[n\right]-{\text{m}}_{i1}^{\left(\text{G}\right)}\left[n\right]. \end{array}$$

The marker-coordinate system of the link-*i* cannot be fixed in the link, due to STA, which is why we call it “pseudo” local coordinate system. Thus, the marker-coordinate system of the link-*i* may exhibit temporal deviations from the landmark-coordinate system, and the relative position between the marker-coordinate system of link-*i* (${\text{o}}_{i}^{\left(\text{G}\right)}\left[n\right]$ and ${\text{A}}_{i}^{\left(\text{G}\right)}\left[n\right]$) and the actual joint position (${\tilde{\text{j}}}^{\left(\text{G}\right)}$) changes in time. Therefore, unlike in the landmark-coordinate system, the joint position vector in the marker-coordinate system ${\text{j}}_{i}^{\left(\text{M-}i\right)}$ inevitably changes in time. Nevertheless, in the naive algorithm, the joint position in the marker-coordinate system of the link-*i* is estimated as the time-independent constant vectors ${\text{j}}_{i}^{\left(\text{M-}i\right)}$ (*i*=A and B) that minimizes the cost function as

(8) $$\begin{array}{r} \underset{{\text{j}}_{\text{A}}^{\left(\text{M-A}\right)},{\text{j}}_{\text{B}}^{\left(\text{M-B}\right)}}{\mathrm{argmin}}\underset{n}{\sum }|\left({\text{o}}_{\text{A}}^{\left(\text{G}\right)}\left[n\right]+{\text{A}}_{\text{A}}^{\left(\text{G}\right)}\left[n\right]{\text{j}}_{\text{A}}^{\left(\text{M-A}\right)}\right)-\left({\text{o}}_{\text{B}}^{\left(\text{G}\right)}\left[n\right]+{\text{A}}_{\text{B}}^{\left(\text{G}\right)}\left[n\right]{\text{j}}_{\text{B}}^{\left(\text{M-B}\right)}\right)|. \end{array}$$

In many cases, there was no pair of constant vectors for the joint positions (${\text{j}}_{\text{A}}^{\left(\text{M-A}\right)}$ and ${\text{j}}_{\text{B}}^{\left(\text{M-B}\right)}$) that make the cost function zero. In other words, the optimal joint position obtained for the time-varying marker-coordinate system for the link-A could not coincide to that for the link-B for all time instances. Thus, in the naive algorithm, the joint position **j**^(G)^[*n*] in the global coordinate system is estimated as the midpoint of the optimal joint positions obtained for each of the two marker-coordinate systems as

(9) $$\begin{array}{r} {\text{j}}^{\left(\text{G}\right)}\left[n\right]=\frac{\left({\text{o}}_{\text{A}}^{\left(\text{G}\right)}\left[n\right]+{\text{A}}_{\text{A}}^{\left(\text{G}\right)}\left[n\right]{\text{j}}_{\text{A}}^{\left(\text{M-A}\right)}\right)+\left({\text{o}}_{\text{B}}^{\left(\text{G}\right)}\left[n\right]+{\text{A}}_{\text{B}}^{\left(\text{G}\right)}\left[n\right]{\text{j}}_{\text{B}}^{\left(\text{M-B}\right)}\right)}{2}. \end{array}$$

The position and the posture of each link are then estimated by using **j**^(G)^[*n*], ${\text{o}}_{i}^{\left(\text{G}\right)}\left[n\right]$, and ${\text{A}}_{\text{i}}^{\left(\text{G}\right)}\left[n\right]$. In this way, the marker positions and the joint position are both affected by STA in the naive algorithm even after the assimilation process, although the assimilated motions of the two-rigid-link model maximally satisfy the joint constraint.

### 2.4. The proposed algorithm for periodic movements

In the algorithm proposed here, unknown positions of the landmark markers and the joint in the global coordinate system are estimated based on the evidence that STA varies periodically during periodic movement, and then the position and the posture of each link are estimated. That is, according to the knowledge that STA profiles during a given periodic motion are also periodic (Inoue et al., 2016), the unknown STA components of the motion-captured marker positions in the unknown landmark-coordinate system are formally represented by a Fourier series. Interestingly, we show that the corresponding Fourier coefficients for the unknown STA can be determined based only on the joint constraint and the periodicity in the distance between two STA-affected markers.

The Fourier coefficients of STA in the landmark-coordinate system is defined first. The STA-affected marker positions in the landmark-coordinate system is denoted as ${\text{m}}_{ij}^{\left(\tilde{\text{M}}\text{-}i\right)}\left[n\right]$. Note that ${\text{m}}_{ij}^{\left(\tilde{\text{M}}\text{-}i\right)}\left[n\right]$ is not the same as ${\tilde{\text{m}}}_{ij}^{\left(\tilde{\text{M}}\text{-}i\right)}\left[n\right]$ that is the position of non-STA-affected landmark marker in the landmark coordinate system. ${\text{m}}_{ij}^{\left(\tilde{\text{M}}\text{-}i\right)}\left[n\right]$ can be described formally as

(10) $$\begin{array}{r} {\text{m}}_{ij}^{\left(\tilde{\text{M}}\text{-}i\right)}\left[n\right]={\left(-1\right)}^{g_{j}}\left[\begin{array}{c} \frac{C_{i}}{2}\\ 0 \end{array}\right]+{\text{e}}_{ij}^{\left(\tilde{\text{M}}\text{-}i\right)}\left[n\right], \end{array}$$

with

$$g_{j}=\{\begin{array}{ll} 1, & \text{for }j=1\\ 2, & \text{for }j=2 \end{array},$$

where *C*_*i*_ is the distance between two landmark markers on the link-*i*, and ${\text{e}}_{ij}^{\left(\tilde{\text{M}}\text{-}i\right)}\left[n\right]$ are the STA profiles represented in the landmark-coordinate system, although we do not know the landmark positions and STA for any instant of time *n*. The first term on the right-hand-side of Equation (10) represents the fixed position of the landmark-*ij* in the landmark-coordinate system of the link-*i*. Since the origin of the landmark-coordinate system is located at the midpoint of two landmarks (Equation 1) and the $x_{i}^{\left(\tilde{\text{M}}\right)}$-axis directs from the landmark marker-*i*1 to the landmark marker-*i*2 (Equation 2), the absolute value of the *x*-component of the first term is one-half of the distance between two landmarks. The *y*-component of the first term is zero by definition of the $y_{i}^{\left(\tilde{\text{M}}\right)}$-axis of the coordinate system.

We expand the STA for the marker-*ij* in the landmark-coordinate system into the following Fourier series:

(11) $$\begin{array}{l} e_{ij}^{\left(\tilde{\text{M}}\text{-}i\right)}[n]=\left[\begin{array}{c} \sum_{k=1}^{K}\left\{a_{ij,x,k}\cos\frac{2\pi kn}{N}+b_{ij,x,k}\sin\frac{2\pi kn}{N}\right\}\\ \sum_{k=1}^{K}\left\{a_{ij,y,k}\cos\frac{2\pi kn}{N}+b_{ij,y,k}\sin\frac{2\pi kn}{N}\right\} \end{array}\right]\\ \;\equiv \left[\begin{array}{cc} P[n] & 0\\ 0 & P[n] \end{array}\right]\left[\begin{array}{c} q_{ij,x}^{\left(\tilde{\text{M}}\text{-}i\right)}\\ q_{ij,y}^{\left(\tilde{\text{M}}\text{-}i\right)} \end{array}\right], \end{array}$$

with

$$\begin{array}{lll} \text{P}\left[n\right] & = & \left[\begin{array}{cccccc} \cos\frac{2\pi 1n}{N} & \ldots & \cos\frac{2\pi Kn}{N} & \sin\frac{2\pi 1n}{N} & \ldots & \sin\frac{2\pi Kn}{N} \end{array}\right],\\ {\text{q}}_{ij,x}^{\left(\tilde{\text{M}}\text{-}i\right)} & = & {\left[\begin{array}{cccccc} a_{ij,x,1} & \ldots & a_{ij,x,K} & b_{ij,x,1} & \ldots & b_{ij,x,K} \end{array}\right]}^{\text{T}},\\ {\text{q}}_{ij,y}^{\left(\tilde{\text{M}}\text{-}i\right)} & = & {\left[\begin{array}{cccccc} a_{ij,y,1} & \ldots & a_{ij,y,K} & b_{ij,y,1} & \ldots & b_{ij,y,K} \end{array}\right]}^{\text{T}}, \end{array}$$

where *N* represents the one cycle data length of the periodic motion, *K* is the order of Fourier series expansion, **P**[*n*] is the row vector composed of the cosine and the sine basis functions, and {*a*_*ij, x, k*_, *b*_*ij, x, k*_} and {*a*_*ij, y, k*_, *b*_*ij, y, k*_} are the Fourier coefficients that define the column vectors ${\text{q}}_{ij,x}^{\left(\tilde{\text{M}}\text{-}i\right)}$ and ${\text{q}}_{ij,y}^{\left(\tilde{\text{M}}\text{-}i\right)}$.

Let ${\text{o}}_{i}^{\left(\tilde{\text{M}}\text{-}i\right)}\left[n\right]$ and ${\text{A}}_{i}^{\left(\tilde{\text{M}}\text{-}i\right)}\left[n\right]$ be the position and the posture of the marker-coordinate system for the link-*i*, respectively (Figure 1D), relative to the landmark-coordinate system (${\tilde{\text{o}}}_{i}^{\left(\text{G}\right)}\left[n\right]$ and ${\tilde{\text{A}}}_{i}^{\left(\text{G}\right)}\left[n\right]$). Using the definition of the marker-coordinate system (Equations 6 , 7) and Equations (10, 11), the excursion of the origin and the tilt of axes of the marker-coordinate system in the landmark-coordinate system, i.e., ${\text{o}}_{i}^{\left(\tilde{\text{M}}\text{-}i\right)}\left[n\right]$ and ${\text{A}}_{i}^{\left(\tilde{\text{M}}\text{-}i\right)}\left[n\right]$, can be expressed by using the Fourier-expanded STA as follows.

(12) $$o_{i}^{\left(\tilde{\text{M}}\text{-}i\right)}[n]=\frac{1}{2}\left[\begin{array}{cc} P[n] & 0\\ 0 & P[n] \end{array}\right]\left[\begin{array}{c} q_{i1,x}^{\left(\tilde{\text{M}}\text{-}i\right)}+q_{i2,x}^{\left(\tilde{\text{M}}\text{-}i\right)}\\ q_{i1,y}^{\left(\tilde{\text{M}}\text{-}i\right)}+q_{i2,y}^{\left(\tilde{\text{M}}\text{-}i\right)} \end{array}\right],$$

(13) $$A_{i}^{\left(\tilde{\text{M}}\text{-}i\right)}[n]=\left[\begin{array}{rr} \cos\theta [n] & -\sin\theta [n]\\ \sin\theta [n] & \cos\theta [n] \end{array}\right],$$

where

(14) $$\begin{array}{r} \theta \left[n\right]=\tan^{-1}\frac{\text{P}\left[n\right]\left({\text{q}}_{i2,y}^{\left(\tilde{\text{M}}\text{-}i\right)}-{\text{q}}_{i1,y}^{\left(\tilde{\text{M}}\text{-}i\right)}\right)}{C_{i}+\text{P}\left[n\right]\left({\text{q}}_{i2,x}^{\left(\tilde{\text{M}}\text{-}i\right)}-{\text{q}}_{i1,x}^{\left(\tilde{\text{M}}\text{-}i\right)}\right)}. \end{array}$$

In this sequel, we show that *C*_*i*_ and the Fourier coefficients of ${\text{e}}_{ij}^{\left(\tilde{\text{M}}\text{-}i\right)}\left[n\right]$ (i.e., STA in the landmark-coordinate system), ${\text{q}}_{ij,x}^{\left(\tilde{\text{M}}\text{-}i\right)}$ and ${\text{q}}_{ij,y}^{\left(\tilde{\text{M}}\text{-}i\right)}$, can be determined based only on the joint constraint and the periodicity-assumption of STA. This means that we can obtain STA-free, corrected time-courses of the marker positions, as ${\text{m}}_{ij}^{\left(\tilde{\text{M}}\text{-}i\right)}\left[n\right]-{\text{e}}_{ij}^{\left(\tilde{\text{M}}\text{-}i\right)}\left[n\right]$ in the landmark-coordinate system, which can be transformed into the landmark-marker positions ${\tilde{\text{m}}}_{ij}^{\left(\text{G}\right)}\left[n\right]$ in the global coordinate system. Once we determine ${\tilde{\text{m}}}_{ij}^{\left(\text{G}\right)}\left[n\right]$ successfully, the position and the posture of each link (${\tilde{\text{o}}}_{i}^{\left(\text{G}\right)}\left[n\right]$ and ${\tilde{\text{A}}}_{i}^{\left(\text{G}\right)}\left[n\right]$), the joint positions ${\tilde{\text{j}}}_{i}^{\left(\tilde{\text{M}}\text{-}i\right)}\left[n\right]$ in the landmark-coordinate system, and the joint position ${\tilde{\text{j}}}^{\left(\text{G}\right)}\left[n\right]$ in the global coordinate system can be obtained using Equations (1, 2, 5), and Equation (3), respectively.

#### 2.4.1. The marker-coordinate system relative to the landmark-coordinate system

Any local vector **u**^(M-*i*)^[*n*] in the marker-coordinate system of the link-*i* can be represented as

(15) $$\begin{array}{r} {\text{u}}^{\left(\tilde{\text{M}}\text{-}i\right)}\left[n\right]={\text{o}}_{i}^{\left(\tilde{\text{M}}\text{-}i\right)}\left[n\right]+{\text{A}}_{i}^{\left(\tilde{\text{M}}\text{-}i\right)}\left[n\right]{\text{u}}^{\left(\text{M-}i\right)}\left[n\right], \end{array}$$

in the landmark-coordinate system, which is also represented in the global coordinate as

(16) $$u^{(\text{G)}}[n]={\tilde{o}}_{i}^{\left(\text{G}\right)}[n]+{\tilde{A}}_{i}^{\left(\text{G}\right)}[n]o_{i}^{\left(\tilde{\text{M}}\text{-}i\right)}[n]+{\tilde{A}}_{i}^{\left(\text{G}\right)}[n]A_{i}^{\left(\tilde{\text{M}}\text{-}i\right)}[n]u^{\left(\text{M-}i\right)}[n]$$

or, alternatively

(17) $$u^{(\text{G)}}[n]=o_{i}^{\left(\text{G}\right)}[n]+A_{i}^{\left(\text{G}\right)}[n]u^{\left(\text{M-}i\right)}[n],$$

by the coordinate transformation directly from the marker-coordinate system (with its origin at ${\text{o}}_{i}^{\left(\text{G}\right)}\left[n\right]$ and the posture ${\text{A}}_{i}^{\left(\text{G}\right)}\left[n\right]$ in the global coordinate system) to the global coordinate system.

Comparing the first and the second terms of the right-hand side of Equation (16) with the first term of the right-hand side in Equation (17), we have

(18) $$\begin{array}{r} {\text{o}}_{i}^{\left(\text{G}\right)}\left[n\right]={\tilde{\text{o}}}_{i}^{\left(\text{G}\right)}\left[n\right]+{\tilde{\text{A}}}_{i}^{\left(\text{G}\right)}\left[n\right]{\text{o}}_{i}^{\left(\tilde{\text{M}}\text{-}i\right)}\left[n\right], \end{array}$$

and comparing the third term of the right-hand side in Equation (16) with the second term of the right-hand side in Equation (17), we have

(19) $$\begin{array}{r} {\text{A}}_{i}^{\left(\text{G}\right)}\left[n\right]={\tilde{\text{A}}}_{i}^{\left(\text{G}\right)}\left[n\right]{\text{A}}_{i}^{\left(\tilde{\text{M}}\text{-}i\right)}\left[n\right]. \end{array}$$

By rearranging Equations (18) and (19), the landmark-coordinate system in the global coordinate system can be related to the marker-coordinate system as

(20) $$\begin{array}{rcl} {\tilde{\text{o}}}_{i}^{\left(\text{G}\right)}\left[n\right] & = & {\text{o}}_{i}^{\left(\text{G}\right)}\left[n\right]-{\text{A}}_{i}^{\left(\text{G}\right)}\left[n\right]{\left({\text{A}}_{i}^{\left(\tilde{\text{M}}\text{-}i\right)}\left[n\right]\right)}^{-1}{\text{o}}_{i}^{\left(\tilde{\text{M}}\text{-}i\right)}\left[n\right], \end{array}$$

(21) $$\begin{array}{rcl} {\tilde{\text{A}}}_{i}^{\left(\text{G}\right)}\left[n\right] & = & {\text{A}}_{i}^{\left(\text{G}\right)}\left[n\right]{\left({\text{A}}_{i}^{\left(\tilde{\text{M}}\text{-}i\right)}\left[n\right]\right)}^{-1}. \end{array}$$

Equations (20) and (21) imply that we can obtain the landmark-coordinate system in the global coordinate system, if, somehow, we can obtain the position and the posture of the marker-coordinate system relative to the landmark-coordinate system, since ${\text{o}}_{i}^{\left(\text{G}\right)}\left[n\right]$ and ${\text{A}}_{i}^{\left(\text{G}\right)}\left[n\right]$ for the marker-coordinate system are available from the motion-captured marker positions using Equations (6) and (7).

#### 2.4.2. The cost function for ensuring the joint constraint

To estimate the Fourier coefficient of STA, ${\text{q}}_{ij,x}^{\left(\tilde{\text{M}}\text{-}i\right)}$ and ${\text{q}}_{ij,y}^{\left(\tilde{\text{M}}\text{-}i\right)}$, a cost function to ensure the joint constraint, such as in Equation (8) for the naive algorithm, is considered also in the proposed algorithm. We show that, through the optimization process for the cost function, a time-invariant constant vector ${\tilde{\text{j}}}_{i}^{\left(\tilde{\text{M}}\text{-}i\right)}$ representing the joint position in the landmark-coordinate system for each link can also be obtained simultaneously together with ${\text{q}}_{ij,x}^{\left(\tilde{\text{M}}\text{-}i\right)}$ and ${\text{q}}_{ij,y}^{\left(\tilde{\text{M}}\text{-}i\right)}$.

Using Equations (20) and (21), the joint position for the link-*i* in the global coordinate system defined by Equation (3) can be rewritten with the constant joint position vector in the landmark-coordinate system ${\tilde{\text{j}}}_{i}^{\left(\tilde{\text{M}}\text{-}i\right)}$ as

(22) $$\begin{array}{l} {\tilde{j}}_{i}^{\left(\text{G}\right)}[n]\\ ={\tilde{o}}_{i}^{\left(\text{G}\right)}[n]+{\tilde{A}}_{i}^{\left(\text{G}\right)}[n]{\tilde{j}}_{i}^{\left(\tilde{\text{M}}\text{-}i\right)}\\ =o_{i}^{\left(\text{G}\right)}[n]-A_{i}^{\left(\text{G}\right)}[n]{\left(A_{i}^{\left(\tilde{\text{M}}\text{-}i\right)}[n]\right)}^{-1}o_{i}^{\left(\tilde{\text{M}}\text{-}i\right)}[n]\\ \;+A_{i}^{\left(\text{G}\right)}[n]{\left(A_{i}^{\left(\tilde{\text{M}}\text{-}i\right)}[n]\right)}^{-1}{\tilde{j}}_{i}^{\left(\tilde{\text{M}}\text{-}i\right)}. \end{array}$$

Reminding that we have ${\text{o}}_{i}^{\left(\text{G}\right)}\left[n\right]$ and ${\text{A}}_{i}^{\left(\text{G}\right)}\left[n\right]$ from the captured marker positions as in Equations (6) and (7), and ${\text{o}}_{i}^{\left(\tilde{\text{M}}\text{-}i\right)}\left[n\right]$ and ${\text{A}}_{i}^{\left(\tilde{\text{M}}\text{-}i\right)}\left[n\right]$ expressed by the Fourier series as in Equations (12) and (13), we now have the landmark-based joint position ${\tilde{\text{j}}}_{i}^{\left(\text{G}\right)}\left[n\right]$ for the link-*i* in the global coordinate system represented as the function of unknown Fourier coefficients of STA ${\text{q}}_{ij,x}^{\left(\tilde{\text{M}}\text{-}i\right)}$ and ${\text{q}}_{ij,y}^{\left(\tilde{\text{M}}\text{-}i\right)}$, the distance between the landmarks *C*_*i*_, as well as ${\tilde{\text{j}}}_{i}^{\left(\tilde{\text{M}}\text{-}i\right)}$.

The joint constraint between the link-A and the link-B is satisfied by an appropriate set of the Fourier coefficients of STA, the distance between landmarks, and the constant joint position vectors in the landmark-coordinate system, if the following function $\tilde{F}$ becomes zero:

(23) $$\begin{array}{r} \tilde{F}=\underset{n}{\sum }|{\tilde{\text{j}}}_{\text{A}}^{\left(\text{G}\right)}\left[n\right]-{\tilde{\text{j}}}_{\text{B}}^{\left(\text{G}\right)}\left[n\right]|. \end{array}$$

In the proposed algorithm, the function $\tilde{F}$ is used as the cost function to be minimized for obtaining the optimal Fourier coefficients of STA, the distance between landmarks, and the joint positions in the landmark-coordinate systems. However, it is not easy to find the optimal parameters that minimize the cost function $\tilde{F}$, because $\tilde{F}$ includes nonlinear terms of the unknown parameters to be solved, such as ${\text{A}}_{i}^{\left(\tilde{\text{M}}\text{-}i\right)}\left[n\right]$ times ${\tilde{\text{j}}}_{i}^{\left(\tilde{\text{M}}\text{-}i\right)}$, both of which are the functions of the unknown parameters. Interestingly, a certain preprocessing, associated with the fact that the distance between two markers on each link should change periodically in time under the periodicity assumption for the STA, will reduce the difficulty in solving the optimization problem. This is a maneuver that makes the proposed algorithm practically useful. We illustrate the maneuver prior to minimizing $\tilde{F}$.

#### 2.4.3. The maneuver for solving the distance between two landmarks for each link and the posture of the marker-coordinate system relative to the landmark-coordinate system

Here we show that *C*_*i*_ (the distance between the landmark-*i*1 and the landmark-*i*2 for the link-*i*) and ${\text{q}}_{i2,x}^{\left(\tilde{\text{M}}\text{-}i\right)}-{\text{q}}_{i1,x}^{\left(\tilde{\text{M}}\text{-}i\right)}$ and ${\text{q}}_{i2,y}^{\left(\tilde{\text{M}}\text{-}i\right)}-{\text{q}}_{i1,y}^{\left(\tilde{\text{M}}\text{-}i\right)}$ (differences between the Fourier coefficients of STA in the landmark-coordinate system of the link-*i*) can be obtained without optimizing the cost function $\tilde{F}$. Then, substituting them into Equation (14), we can obtain the posture of the marker-coordinate system ${\text{A}}_{i}^{\left(\tilde{\text{M}}\text{-}i\right)}\left[n\right]$ relative to the landmark-coordinate system.

To this end, we utilize the following identity,

(24) $$\begin{array}{r} |{\text{m}}_{i2}^{\left(\tilde{\text{M}}\text{-}i\right)}\left[n\right]-{\text{m}}_{i1}^{\left(\tilde{\text{M}}\text{-}i\right)}\left[n\right]|=|{\text{m}}_{i2}^{\left(\text{G}\right)}\left[n\right]-{\text{m}}_{i1}^{\left(\text{G}\right)}\left[n\right]|, \end{array}$$

which expresses the fact that the distance between two motion-captured markers on the link-*i* in the landmark-coordinate system (the left-hand-side of the identity) and the distance between those in the global coordinate system (the right-hand-side of the identity) are independent of the coordinate system, and they are identical for any instant of time *n*, although the distance may change periodically in time due to the periodicity of STA.

For the left-hand-side of Equation (24), we rewrite the square of it using Equations (10) and (11), and differences between vectors of Fourier coefficients of STA and *C*_*i*_, according to the Pythagorean theorem, as follows.

(25) $$\begin{array}{r} |{\text{m}}_{i2}^{\left(\tilde{\text{M}}\text{-}i\right)}\left[n\right]-{\text{m}}_{i1}^{\left(\tilde{\text{M}}\text{-}i\right)}\left[n\right]{|}^{2}={\left(C_{i}+\text{P}\left[n\right]\xi_{i,x}^{\left(\tilde{\text{M}}\text{-}i\right)}\right)}^{2}+{\left(\text{P}\left[n\right]\xi_{i,y}^{\left(\tilde{\text{M}}\text{-}i\right)}\right)}^{2}, \end{array}$$

where

$$\begin{array}{l} {\text{ξ}}_{i,x}^{\left(\tilde{\text{M}}\text{-}i\right)}=q_{i2,x}^{\left(\tilde{\text{M}}\text{-}i\right)}-q_{i1,x}^{\left(\tilde{\text{M}}\text{-}i\right)},\\ {\text{ξ}}_{i,y}^{\left(\tilde{\text{M}}\text{-}i\right)}=q_{i2,y}^{\left(\tilde{\text{M}}\text{-}i\right)}-q_{i1,y}^{\left(\tilde{\text{M}}\text{-}i\right)}. \end{array}$$

That is, ${\text{ξ}}_{i,x}^{\left(\tilde{\text{M}}\text{-}i\right)}$ and ${\text{ξ}}_{i,y}^{\left(\tilde{\text{M}}\text{-}i\right)}$ represent the differences between the Fourier coefficients of STA in the landmark-coordinate system. Note that, by expanding the square terms in the right-hand-side of Equation (25) concretely, and utilizing the product-to-sum formulae for sine and cosine functions of the Fourier series, the right-hand-side of Equation (25) can be expressed as the sum of terms in the form of *v*_*i, k*_cos(2π*kn*/*N*) and *w*_*i, k*_sin(2π*kn*/*N*), where *k* runs from 1 to 2*K*, with an additional constant term $C_{i}^{\prime }$. Note also that, in this expanded form of Equation (25), *v*_*i, k*_, *w*_*i, k*_, and $C_{i}^{\prime }$ are the functions of ${\text{ξ}}_{i,x}^{\left(\tilde{\text{M}}\text{-}i\right)}$, ${\text{ξ}}_{i,y}^{\left(\tilde{\text{M}}\text{-}i\right)}$, and *C*_*i*_.

For the right-hand-side of Equation (24), we expand the square of it by another Fourier series as

(26) $${\left|m_{i2}^{\left(\text{G}\right)}[n]-m_{i1}^{\left(\text{G}\right)}[n]\right|}^{2}=\gamma_{i}+\sum_{k=1}^{2K}\left\{\alpha_{i,k}\cos\frac{2\pi kn}{N}+\beta_{i,k}\sin\frac{2\pi kn}{N}\right\},$$

which is possible because temporal changes in the distance between two markers in the global coordinate system are also periodic by the periodicity assumption for STA. It is important to note that the left-hand-side of Equation (27) can be obtained directly from the motion-captured data ${\text{m}}_{ij}^{\left(\text{G}\right)}\left[n\right]$, which can easily be Fourier-expanded to obtain the coefficients α_*i, k*_, β_*i, k*_, and γ_*i*_.

Then the identity between the right-hand-side of Equation (25) and that of Equation (27) can be guaranteed by term-wise equalization for the coefficients of cos(2π*kn*/*N*), (i.e., *v*_*i, k*_ = α_*i, k*_) and those of sin(2π*kn*/*N*), (i.e., *w*_*i, k*_ = β_*i, k*_) for *k* = 1, 2, ⋯ , 2*K*. The unknown parameters $\xi_{i,x}^{\left(\tilde{\text{M}}\text{-}i\right)}$, $\xi_{i,y}^{\left(\tilde{\text{M}}\text{-}i\right)}$, and *C*_*i*_ can be solved from the 4*K*+1 set of equations, since *v*_*i, k*_ and *w*_*i, k*_ are the quadratic functions of those unknown. Substituting the solved parameter values of the difference in the Fourier coefficients of STA, namely $\xi_{i,x}^{\left(\tilde{\text{M}}\text{-}i\right)}={\text{q}}_{i2,x}^{\left(\tilde{\text{M}}\text{-}i\right)}-{\text{q}}_{i1,x}^{\left(\tilde{\text{M}}\text{-}i\right)}$, $\xi_{i,y}^{\left(\tilde{\text{M}}\text{-}i\right)}={\text{q}}_{i2,y}^{\left(\tilde{\text{M}}\text{-}i\right)}-{\text{q}}_{i1,y}^{\left(\tilde{\text{M}}\text{-}i\right)}$, and *C*_*i*_ into Equation (14), we obtain the posture of the marker-coordinate system ${\text{A}}_{i}^{\left(\tilde{\text{M}}\text{-}i\right)}\left[n\right]$ relative to the landmark-coordinate system.

#### 2.4.4. Solving the positions of the landmarks and the joint in the global coordinate system by minimizing the cost function

So far, we have obtained the differences in the Fourier coefficients of STA ($\xi_{i,x}^{\left(\tilde{\text{M}}\text{-}i\right)}$ and $\xi_{i,y}^{\left(\tilde{\text{M}}\text{-}i\right)}$) and the posture of the marker-coordinate system (${\text{A}}_{i}^{\left(\tilde{\text{M}}\text{-}i\right)}\left[n\right]$) relative to the landmark-coordinate system. Those solutions make the optimization of the cost function $\tilde{F}$ defined by Equation (23) easy, as shown in this sequel. Indeed, one can confirm that the cost function can be rewritten as follows.

(27) $$\begin{array}{l} \tilde{F}=\sum_{n}|\{o_{\text{A}}^{\left(\text{G}\right)}[n]-\frac{1}{2}B_{\text{A}}[n]\left[\begin{array}{c} {\text{ξ}}_{A,x}^{\left(\tilde{\text{M}}\text{-A}\right)}\\ {\text{ξ}}_{A,y}^{\left(\tilde{\text{M}}\text{-A}\right)} \end{array}\right]-B_{\text{A}}[n]\left[\begin{array}{c} q_{\text{A}1,x}^{\left(\tilde{\text{M}}\text{-A}\right)}\\ q_{\text{A}1,y}^{\left(\tilde{\text{M}}\text{-A}\right)} \end{array}\right]\\ \;+{\tilde{A}}_{\text{A}}^{\left(\text{G}\right)}[n]{\tilde{j}}_{\text{A}}^{\left(\tilde{\text{M}}\text{-A}\right)}\}-\{o_{\text{B}}^{\left(\text{G}\right)}[n]-\frac{1}{2}B_{\text{B}}[n]\left[\begin{array}{c} {\text{ξ}}_{B,x}^{{}^{\left(\tilde{\text{M}}\text{-B}\right)}}\\ {\text{ξ}}_{B,y}^{\left(\tilde{\text{M}}\text{-B}\right)} \end{array}\right]\\ \;-B_{\text{B}}[n]\left[\begin{array}{c} q_{\text{B}1,x}^{\left(\tilde{\text{M}}\text{-B}\right)}\\ q_{\text{B}1,y}^{\left(\tilde{\text{M}}\text{-B}\right)} \end{array}\right]+{\tilde{A}}_{\text{B}}^{\left(\text{G}\right)}[n]{\tilde{j}}_{\text{B}}^{\left(\tilde{\text{M}}\text{-B}\right)}\}|, \end{array}$$

where

$$B_{i}[n]={\tilde{A}}_{i}^{\left(\text{G}\right)}[n]\left[\begin{array}{cc} P[n] & 0\\ 0 & P[n] \end{array}\right].$$

In Equation (28), the unknown parameters that should minimize the cost are reduced only to the Fourier coefficients of STA for the marker-*i*1 (${\text{q}}_{i1,x}^{\left(\tilde{\text{M}}\text{-}i\right)}$ and ${\text{q}}_{i1,y}^{\left(\tilde{\text{M}}\text{-}i\right)}$) and the time-invariant constant vector of the joint position in the landmark-coordinate system (${\tilde{\text{j}}}_{i}^{\left(\tilde{\text{M}}\text{-}i\right)}$). Since $\tilde{F}$ is now a linear function of those unknown parameters, the optimal values of those parameters can be obtained easily as

(28) $$\begin{array}{r} \underset{{\text{q}}_{i1,x}^{\left(\tilde{\text{M}}\text{-}i\right)},{\text{q}}_{i1,y}^{\left(\tilde{\text{M}}\text{-}i\right)},{\tilde{\text{j}}}_{i}^{\left(\tilde{\text{M}}\text{-}i\right)}}{\mathrm{argmin}}\tilde{F}. \end{array}$$

Once we have ${\text{q}}_{i1,x}^{\left(\tilde{\text{M}}\text{-}i\right)}$ and ${\text{q}}_{i1,y}^{\left(\tilde{\text{M}}\text{-}i\right)}$, it follows simply that ${\text{q}}_{i2,x}^{\left(\tilde{\text{M}}\text{-}i\right)}=\xi_{i,x}^{\left(\tilde{\text{M}}\text{-}i\right)}+{\text{q}}_{i1,x}^{\left(\tilde{\text{M}}\text{-}i\right)}$ and ${\text{q}}_{i2,y}^{\left(\tilde{\text{M}}\text{-}i\right)}=\xi_{i,y}^{\left(\tilde{\text{M}}\text{-}i\right)}+{\text{q}}_{i1,y}^{\left(\tilde{\text{M}}\text{-}i\right)}$. In this way, we could successfully determine the STA in the landmark-coordinate system as the Fourier series of Equation (11), as well as the joint position in each of the landmark-coordinate systems for the link-A and the link-B.

Using the Fourier coefficients of STA in the landmark-coordinate system and the distance between two landmarks for each link, the position and the posture of the landmark-coordinate system in the global coordinate system (${\tilde{\text{o}}}_{i}^{\left(\text{G}\right)}\left[n\right]$ and ${\tilde{\text{A}}}_{i}^{\left(\text{G}\right)}\left[n\right]$) can also be obtained directly from Equations (20) and (21). Moreover, the position of the landmarks and the joint in the global coordinate system (${\tilde{\text{m}}}_{ij}^{\left(\text{G}\right)}\left[n\right]$ and ${\tilde{\text{j}}}_{i}^{\left(\text{G}\right)}\left[n\right]$) can be calculated using Equations (10) and (22). As in the case with the naive algorithm, the joint position is estimated as the average of ${\tilde{\text{j}}}_{\text{A}}^{\left(\text{G}\right)}$ and ${\tilde{\text{j}}}_{\text{B}}^{\left(\text{G}\right)}$ as follows.

(29) $$\begin{array}{r} {\tilde{\text{j}}}^{\left(\text{G}\right)}\left[n\right]=\frac{{\tilde{\text{o}}}_{\text{A}}^{\left(\text{G}\right)}\left[n\right]+{\tilde{\text{A}}}_{\text{A}}^{\left(\text{G}\right)}\left[n\right]{\tilde{\text{j}}}_{\text{A}}^{\left(\tilde{\text{M}}\text{-A}\right)}+{\tilde{\text{o}}}_{\text{B}}^{\left(\text{G}\right)}\left[n\right]+{\tilde{\text{A}}}_{\text{B}}^{\left(\text{G}\right)}\left[n\right]{\tilde{\text{j}}}_{\text{B}}^{\left(\tilde{\text{M}}\text{-B}\right)}}{2} \end{array}$$

From these positions, we can obtain the exact position and posture of the link-coordinate system for each link.

## 3. Methods for evaluating the proposed algorithm

**Figure 2:**
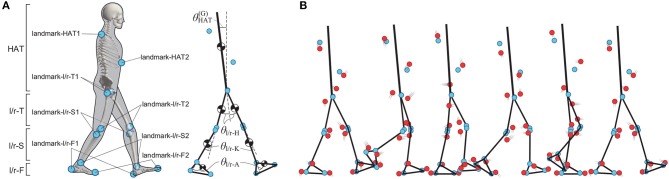
Rigid seven-link model of human walking. **(A)** Positions of landmarks and rigid seven-link model of human body. Rigid seven-link model consists of Head-Arm-Trunk link (HAT), left and right Thigh links (l/r-T), left and right Shank links (l/r-S), and left and right Foot links (l/r-F). Blue circles represent landmarks of each link, and each landmark corresponds to anatomical landmark of human body as follows: landmark-HAT1 is seventh cervical vertebra, landmark-HAT2 is xiphoid process, landmark-l/r-T1 is greater trochanter, landmark-l/r-T2 is lateral condyle of femur, landmark-l/r-S1 is lateral condyle of tibia, landmark-l/r-S2 is lateral malleolus, landmark-l/r-F1 is calcaneus, and landmark-l/r-F2 is first Metatarsal bone. $\theta_{\text{HAT}}^{\left(\text{G}\right)}$ is posture of HAT link, which is represented as tilt angle from vertical axis. Joint angles at left/right-Hip (θ_l/r-H_), left/right-Knee (θ_l/r-K_), and left/right-Ankle (θ_l/r-A_), are defined as relative angle for the proximal link. These angles take positive value for clockwise rotation, and negative value for counterclockwise rotation. See Table 1 for details of variables and parameters of the model. **(B)** Conceptual diagram of methods for evaluating the proposed algorithm. Black lines show walking dynamics of rigid link model, and blue circles are landmarks which move with the rigid link model. Red circles are captured markers which excurse due to STA. See main text for details.

To assess the efficiency of the proposed algorithm, we conducted a numerical experiment using a rigid seven-link model that moves in the sagittal plane (Figure 2), as utilized in Yamasaki et al. (2003). The model consists of a head-arm-trunk (HAT) link, left and right thigh links (l/r-T), left and right shank links (l/r-S), and left and right foot links (l/r-F), which are connected by the pin joints (Figure 2A). See Table 1 for dynamic variables and parameters of the model. Two landmarks (and two landmark-markers), i.e., the landmark-*i*1 and the landmark-*i*2 for *i*={HAT,l/r-T,l/r-S,l/r-F} fixed on each link are defined as in Figure 2A. In this experiment, we set the link-coordinate system for each link such that the origin is located at the CoM of the link and the $x_{i}^{\left(\text{L}\right)}$-axis directs from the CoM to the distal end of the link. For a given set of two landmarks for each link, position and posture of the link-coordinate system can be determined by the centroid of the triangle having the vertices at the joint and two landmarks as in section 2.2, since the CoM position may be available from the statistics of body-parameters, and thus the relative position of the CoM and the centroid can be obtained.

**Table 1:** Variables and parameters of the rigid seven-link model.

**Symbol**	**Description**	****Value/Unit****
$\theta_{\text{HAT}}^{\left(\text{G}\right)}$	Posture of Head-Arm-Trunk link	— rad
θ_l/r-H_	Angular degree of left/right-Hip joint	— rad
θ_l/r-K_	Angular degree of left/right-Knee joint	— rad
θ_l/r-A_	Angular degree of left/right-Ankle joint	— rad
τ_l/r-H_	Torque of left/right-Hip joint	— Nm
τ_l/r-K_	Torque of left/right-Knee joint	— Nm
τ_l/r-A_	Torque of left/right-Ankle joint	— Nm
*m*_HAT_	Mass of Head-Arm-Trunk link	40.548 kg
*m*_Thigh_	Mass of Thigh link	6.882 kg
*m*_Shank_	Mass of Shank link	3.162 kg
*m*_Foot_	Mass of Foot link	0.682 kg
*l*_HAT_	Length of Head-Arm-Trunk link	0.536 m
*l*_Thigh_	Length of Thigh link	0.420 m
*l*_Shank_	Length of Shank link	0.379 m
*l*_Foot_	Length of Foot link	0.122 m
*d*_HAT-Hip_	Distance between CoM of HAT and Hip joint	0.204 m
*d*_Hip-Thigh_	Distance between CoM of Thigh and Hip joint	0.200 m
*d*_Knee-Shank_	Distance between CoM of Shank and Knee joint	0.154 m
*d*_Ankle-Foot_	Distance between CoM of Foot and Ankle joint	0.050 m
*d*_Ankle-Heel_	Distance between Ankle and Heel	0.079 m
*I*_HAT_	Inertia moment of Head-Arm-Trunk link	1.09933 m
*I*_Thigh_	Inertia moment of Thigh link	0.09485 m
*I*_Shank_	Inertia moment of Shank link	0.03001 m
*I*_Foot_	Inertia moment of Foot link	0.00014 m

First, we performed dynamic simulations (forward-dynamics) of the model to obtain stable steady-state periodic walking and the corresponding kinematics of each link, as well as two landmark-markers fixed on each link, i.e., 14 markers in total (Figure 2B). See Yamasaki et al. (2003) for details of the simulation. Specifically, from the simulated kinematics, we obtained time-series of the position and the posture of HAT link (${\tilde{\text{o}}}_{\text{HAT}}^{\left(\text{G}\right)}\left[n\right]$ and $\theta_{\text{HAT}}^{\left(\text{G}\right)}\left[n\right]$) and six joint angles of θ_*m*_[*n*] for *m*={l/r-H,l/r-K,l/r-A} with the subscript *m* specifying the joint (not the link) as the left/right hip joint (l/r-H), the left/right knee joint (l/r-K), and the left/right ankle joint (l/r-A). We then calculated the position and the posture of the landmark-coordinate system of each link in the global coordinate system, i.e., ${\tilde{\text{o}}}_{i}^{\left(\text{G}\right)}\left[n\right]$ and ${\tilde{\text{A}}}_{i}^{\left(\text{G}\right)}\left[n\right]$ for *i*={HAT,l/r-T,l/r-S,l/r-F}, referred to as *the true kinematics*, which we would like to retrieve from motion data of STA-affected markers in this experiment. Note that the link-*i* for *i*={l/r-T,l/r-S} is connected to two adjacent links at proximal and distal joints in this model, unlike in the simple two-link model used in section 2, and thus the symbol ${\text{j}}_{i}^{\left(\text{M-}i\right)}\left[n\right]$ used for the joint position cannot specify the joint uniquely, whether it is proximal or distal joint of the link-*i*. To resolve this situation, the position of each joint in a given coordinate system is specified with the joint-name subscript *m*, such as ${\text{j}}_{i,m}^{\left(\text{M-}i\right)}\left[n\right]$ for *m*={l/r-H,l/r-K,l/r-A}, meaning that, in this case, the position of the *m*-th joint in the marker-coordinate system of the link-*i*. For example, ${\text{j}}_{\text{r-T,r-H}}^{\left(\text{M-T}\right)}\left[n\right]$ and ${\text{j}}_{\text{r-T,r-K}}^{\left(\text{M-T}\right)}\left[n\right]$ can be distinguished, respectively, as the position of the right hip joint in the marker-coordinate system of the right thigh and that of the right knee joint also in the marker-coordinate system of the right thigh.

**Figure 3:**
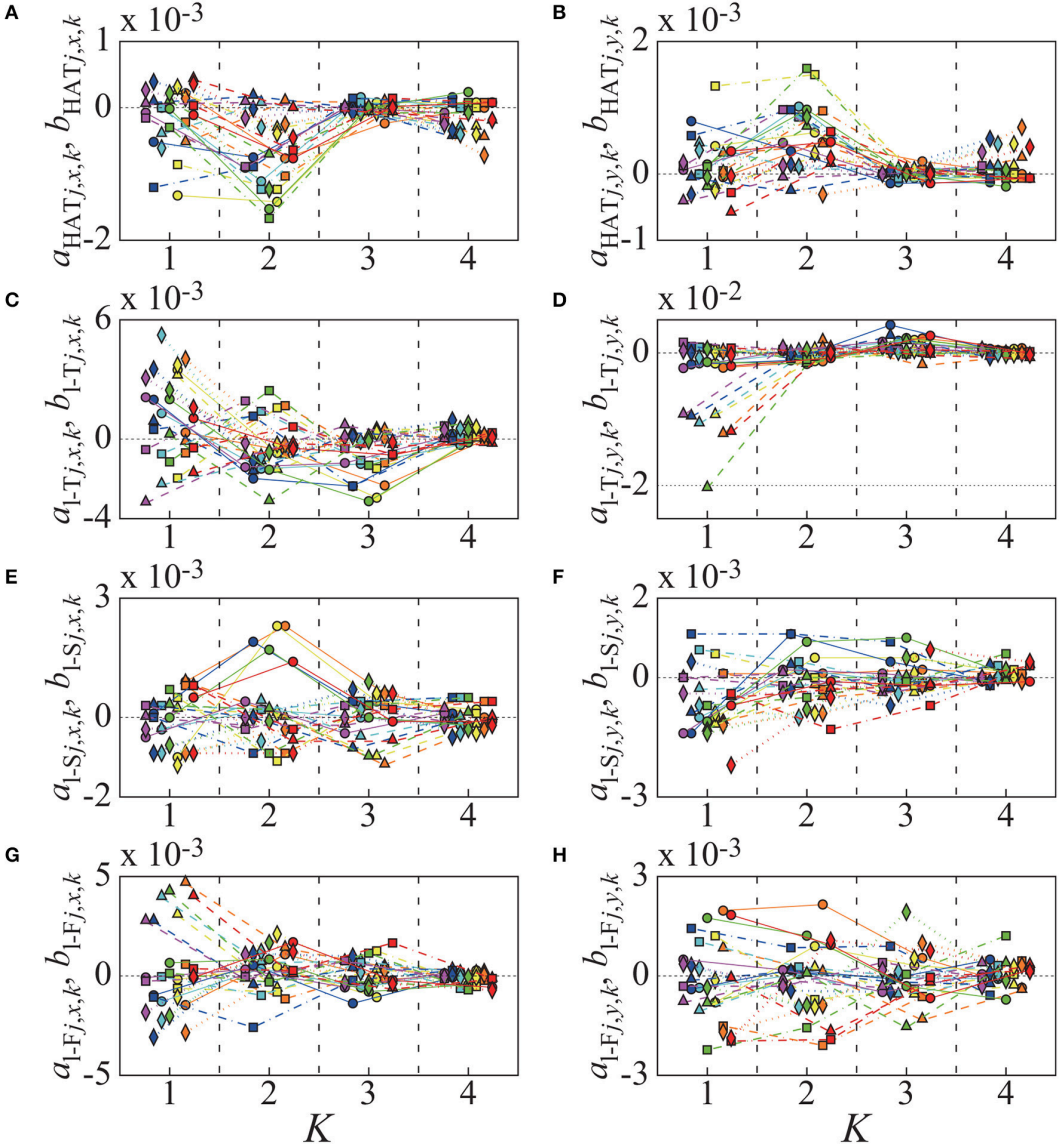
Fourier coefficients of STA of markers, used in the numerical experiments for assessment of efficiency of the new posture estimation algorithm (Equation 31). These coefficients were obtained from experimental measurement data in our previous work, using old algorithm (“naive algorithm,” Inoue et al., 2016). We obtained the coefficients from seven subjects, and different color indicates different subject (purple: sub 1, blue: sub 2, aqua: sub 3, green: sub 4, yellow: sub 5, orange: sub 6, red: sub 7). Circles connected with solid lines are Fourier coefficients of sine terms STA of marker-*i*1 (*a*_*i*1, *x, k*_, *a*_*i*1, *y, k*_), triangles with dashed lines are those of cosine terms of STA of marker-*i*1 (*b*_*i*1, *x, k*_, *b*_*i*1, *y, k*_), tetragons with chain lines are those of sine terms of marker-*i*2 (*a*_*i*2, *x, k*_, *a*_*i*2, *y, k*_), and diamonds with dotted lines are those of cosine terms of marker-*i*2 (*b*_*i*2, *x, k*_, *b*_*i*2, *y, k*_). **(A,B)** Fourier coefficients of *x*-elements and *y*-elements of STA of markers, which are attached on HAT. **(C,D)** Those of markers on left Thigh. **(E,F)** Those of markers on left Shank. **(G,H)** Those of markers on left Foot.

Subsequently, we introduced STA-affected marker positions in the same dynamic simulation as above (Figure 2B). In our previous study, we characterized temporal changes in STA-affected markers that were experimentally motion-captured from seven subjects during periodic walking, in which the markers were attached on the landmarks corresponding to the landmark-*ij* in Figure 2A (Inoue et al., 2016). Moreover, we obtained a rough estimation of the STA profile for each of seven links in the marker-coordinate system using the naive algorithm. We showed that the STA in the marker-coordinate system for each link might be periodic, and could be fitted by the fourth order Fourier series of the gait cycle. In this study, we considered those STA estimates [i.e., the Fourier coefficients (*a*_*ij, x, k*_, *b*_*ij, x, k*_) and (*a*_*ij, y, k*_, *b*_*ij, y, k*_) as in Equation (11) for *k* = 1, ⋯ , 4 (i.e., *K* = 4)] obtained in the previous study as simulated STA profiles in the landmark-coordinate system. Figure 3 shows the Fourier coefficients (*a*_*ij, x, k*_, *b*_*ij, x, k*_) and (*a*_*ij, y, k*_, *b*_*ij, y, k*_) of those STA profiles. As confirmed in Figure 3, the Fourier coefficients of each marker are qualitatively similar across subjects, but also exhibit some individual characteristics quantitatively. It should be noted that those simulated STA profiles used in this experiment were not the “true STA,” because they were estimated by the naive algorithm in the marker-coordinate system, not in the link-coordinate, nor the landmark-coordinate systems. However, we assumed that the STAs estimated by the naive algorithm might be qualitatively similar to the true STA, and they might be good enough to examine performance of the proposed algorithm.

The simulated marker positions ${\text{m}}_{ij}^{\left(\text{G}\right)}\left[n\right]$ in the global coordinate system (corresponding to motion-captured positions of the STA-affected markers) for the *j*-th marker of the link-*i* were represented by the Fourier-expanded STA and the kinematic data of the model (${\tilde{\text{o}}}_{i}^{\left(\text{G}\right)}\left[n\right]$ and ${\tilde{\text{A}}}_{i}^{\left(\text{G}\right)}\left[n\right]$) as follows.

(30) $$\begin{array}{l} m_{ij}^{\left(\text{G}\right)}[n]\\ ={\tilde{o}}_{i}^{\left(\text{G}\right)}[n]\\ +\;{\tilde{A}}_{i}^{\left(\text{G}\right)}[n]\left\{{\tilde{m}}_{ij}^{\left(\tilde{\text{M}}\text{-}i\right)}+\left[\begin{array}{c} \sum_{k=1}^{4}\left\{a_{ij,x,k}\cos\frac{2\pi kn}{N}+b_{ij,x,k}\sin\frac{2\pi kn}{N}\right\}\\ \sum_{k=1}^{4}\left\{a_{ij,y,k}\cos\frac{2\pi kn}{N}+b_{ij,y,k}\sin\frac{2\pi kn}{N}\right\} \end{array}\text{​​}\right]\right\}, \end{array}$$

where ${\tilde{\text{m}}}_{ij}^{\left(\tilde{\text{M}}\text{-}i\right)}$ is the landmark marker position in the landmark-coordinate system of the link-*i*. Since this is a simulated examination of the algorithm, we know all of the parameters in this equation, but we consider a situation that we have a set of time-series data for the STA-affected ${\text{m}}_{ij}^{\left(\text{G}\right)}\left[n\right]$ only, and the others, i.e., ${\tilde{\text{o}}}_{i}^{\left(\text{G}\right)}\left[n\right]$, ${\tilde{\text{A}}}_{i}^{\left(\text{G}\right)}\left[n\right]$, ${\tilde{\text{m}}}_{ij}^{\left(\tilde{\text{M}}\text{-}i\right)}$, and the STA, are all unknown. The result section shows that we could estimate all of those unknowns from the simulated STA-affected marker positions ${\text{m}}_{ij}^{\left(\text{G}\right)}\left[n\right]$ using the proposed algorithm.

### 3.1. Gait data assimilation by the naive and proposed algorithms

#### 3.1.1. Assimilation by the naive algorithm for comparison

In the naive algorithm, position and posture of the marker-coordinate system in the global coordinate system ${\text{o}}_{i}^{\left(\text{G}\right)}\left[n\right]$ and ${\text{A}}_{i}^{\left(\text{G}\right)}\left[n\right]$ for the link-*i* were calculated from the marker positions by Equations (6) and (7). Position of the joint-*m*, ${\text{j}}_{i_{1},m}^{\left(\text{M-}i_{1}\right)}\left[n\right]$, in the marker-coordinate system of the link-*i*_1_ and ${\text{j}}_{i_{2},m}^{\left(\text{M-}i_{2}\right)}\left[n\right]$ in the marker-coordinate system of the link-*i*_2_ were estimated by Equation (8) for two links *i*_1_ and *i*_2_ that are connected at the joint *m*. The corresponding joint position **j**^(G)^[*n*] for the joint *m* in the global coordinate system was then calculated from ${\text{o}}_{i_{1}}^{\left(\text{G}\right)}\left[n\right]$, ${\text{o}}_{i_{2}}^{\left(\text{G}\right)}\left[n\right]$, ${\text{A}}_{i_{1}}^{\left(\text{G}\right)}\left[n\right]$, ${\text{A}}_{i_{2}}^{\left(\text{G}\right)}\left[n\right]$, ${\text{j}}_{i_{1},m}^{\left(\text{M-}i_{1}\right)}$, and ${\text{j}}_{i_{2},m}^{\left(\text{M-}i_{2}\right)}$ by Equation (9). Finally, we retrieved the link-coordinate systems ${\bar{\text{o}}}_{i}^{\left(\text{G}\right)}\left[n\right]$ located at the CoM of the link-*i* and ${\bar{\text{A}}}_{i}^{\left(\text{G}\right)}\left[n\right]$ for all *i*, and then obtained the estimates of the joint angles θ_*m*_[*n*] of the joint-*m* for all *m*.

#### 3.1.2. Assimilation by the proposed algorithm

In the proposed algorithm, we first determined the distance between two landmarks *C*_*i*_ and the differences between Fourier coefficients of STA $\xi_{i,x}^{\left(\tilde{\text{M}}\text{-}i\right)}$ and $\xi_{i,y}^{\left(\tilde{\text{M}}\text{-}i\right)}$, defined by Equations (10), (11), and (25), for each of the link-*i* from the STA-affected marker positions ${\text{m}}_{ij}^{\left(\text{G}\right)}\left[n\right]$. In this process, we used function *fsolve* in MATLAB to find a solution of the quadratic simultaneous equations derived from Equations (25) and (27). An initial condition for the searching difference between the Fourier coefficients in *fsolve* was set to the Fourier coefficients of STA obtained by the naive algorithm (see Inoue et al., 2016 for details).

Subsequently, we estimated the Fourier coefficients ${\text{q}}_{ij,x}^{\left(\tilde{\text{M}}\text{-}i\right)}$, ${\text{q}}_{ij,y}^{\left(\tilde{\text{M}}\text{-}i\right)}$, and the joint position ${\tilde{\text{j}}}_{i,m}^{\left(\tilde{\text{M}}\text{-}i\right)}$ in the landmark-coordinate system using *C*_*i*_, $\xi_{i,x}^{\left(\tilde{\text{M}}\text{-}i\right)}$ and $\xi_{i,y}^{\left(\tilde{\text{M}}\text{-}i\right)}$ by minimizing the cost function $\tilde{F}$ of Equation (28) as Equation (29). We then determined the position ${\tilde{\text{o}}}_{i}^{\left(\text{G}\right)}\left[n\right]$ and the posture ${\tilde{\text{A}}}_{i}^{\left(\text{G}\right)}\left[n\right]$ of the landmark-coordinate system for each link using Equations (20) and (21), from which we calculated the positions of the landmarks ${\tilde{\text{m}}}_{ij}^{\left(\text{G}\right)}\left[n\right]$ and the joints ${\tilde{\text{j}}}_{i,m}^{\left(\text{G}\right)}\left[n\right]$ in the global coordinate system using ${\tilde{\text{j}}}_{i,m}^{\left(\tilde{\text{M}}\text{-}i\right)}$, Equations (10) and (22), and then ${\tilde{\text{j}}}_{m}^{\left(\text{G}\right)}\left[n\right]$ by Equation (30). Finally, we retrieved the link-coordinate systems ${\bar{\text{o}}}_{i}^{\left(\text{G}\right)}\left[n\right]$ located at the CoM of the link-*i* and ${\bar{\text{A}}}_{i}^{\left(\text{G}\right)}\left[n\right]$ for all *i*, and then obtained the assimilated joint angles θ_*m*_[*n*] of the joint-*m* for all *m*.

### 3.2. Comparison between the naive and the proposed algorithms

We compared the assimilated posture of HAT link $\theta_{\text{HAT}}^{\left(\text{G}\right)}\left[n\right]$ and the joint angles θ_*m*_[*n*] by the naive and the proposed algorithms with the true kinematics. Moreover, we examined length of each link, i.e., the distance between proximal and distal joints that were estimated by each of two algorithms. Due to the STA, the origin of the link-coordinate system and the joint position in the global coordinate system for each link assimilated by the naive and the proposed algorithms could include errors, which might induce errors in the link-length. To examine those errors, we calculated the lengths of the thigh and the shank links as the distances between the associated joints, and for those of HAT and the foot links, the distances between the origin of the link-coordinate system (the CoM position) and the distal or the proximal joint were calculated as the functions of time for one gait cycle. The obtained mean length of each link was used in the following inverse dynamics analysis.

#### 3.2.1. Comparison in the inverse dynamics analysis

We performed the inverse dynamics analysis to compare errors in the joint torques based on the assimilated HAT and the six joint angles by the naive and the proposed algorithms with the true joint torques. The true joint torques ${\tilde{\tau }}_{m}$ for the joint-*m* were obtained from the true kinematics, the corresponding ground reaction forces that were obtained in the forward dynamics simulation, and the model parameters shown in Table 1 (see Appendix A for details of the inverse dynamics analysis).

Estimations of the joint torques, denoted by τ_*m*_, were also calculated for two assimilated motions by the naive and the proposed algorithms, in which we used the estimated joint angles and the estimated length of each link that had been obtained in the assimilation process as described above. We used the true values for the mass and the inertia moment for each link, and for the ground reaction forces. We then compared the joint torque τ_*m*_ with the true joint torque ${\tilde{\tau }}_{m}$ for all *m* to assess the efficiency of the proposed algorithm.

## 4. Results of the evaluation

**Figure 4:**
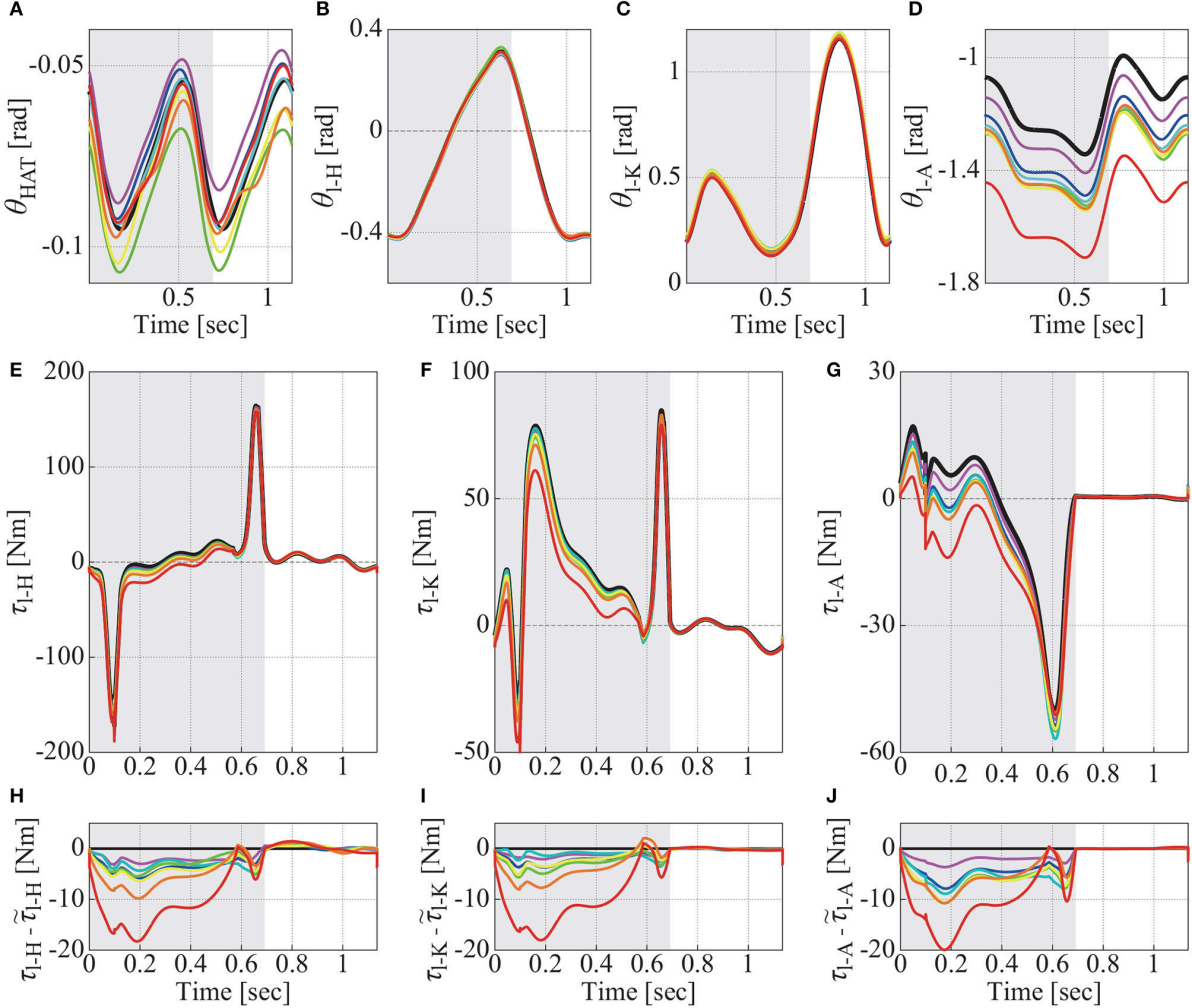
Kinematics and joint torques estimated from spatio-temporal data of markers including STA, which correspond to Fourier coefficients shown in Figure 3. In each panel, there are seven colored curves and one black curve. Colored curves in **(A–D)** are estimated kinematics by using naive algorithm from simulated marker positions with STA, of which Fourier coefficients are plotted with same color in Figure 3. Those curves in **(E–G)** are joint torques calculated from the estimated kinematics (τ_l-H_, τ_l-K_, τ_l-A_), and those in **(H–J)** are difference between the calculated joint torques and joint torques of original kinematics (${\tilde{\tau }}_{\text{l-H}}$, ${\tilde{\tau }}_{\text{l-K}}$, ${\tilde{\tau }}_{\text{l-A}}$). Black curve in each panel shows kinematics and joint torques, which were estimated by using new algorithm proposed in this study. Since the proposed algorithm estimated model kinematics that was exactly same with original kinematics for all (=seven) case of STA, only one curve are described in each panel, and black straight lines at zero are drawn in **(H–J)**. Gray shaded region in each panel represents duration when left Foot contacts ground (stance phase of left leg). **(A)** Postures of HAT link ($\theta_{\text{HAT}}^{\left(\text{G}\right)}$). **(B)** Joint angles of left-Hip (θ_l-H_). **(C)** Those of left-Knee (θ_l-K_). **(D)** Those of left Ankle (θ_l-A_). **(E)** Joint torques at left-Hip (τ_l-H_). **(F)** Those at left-Knee (τ_l-K_). **(G)** Those at left-Ankle (τ_l-A_). **(H)** Difference between joint torques at left-Hip ($\tau_{\text{l-H}}-{\tilde{\tau }}_{\text{l-H}}$). **(I)** Those at left-Knee ($\tau_{\text{l-K}}-{\tilde{\tau }}_{\text{l-K}}$). **(J)** Those at left-Ankle ($\tau_{\text{l-A}}-{\tilde{\tau }}_{\text{l-A}}$).

In the numerical experiment, we successfully simulated a stable periodic sequence of gait and the corresponding true kinematics, from which we could obtain the STA-affected marker positions for each of seven motion-captured subjects shown in Figure 3. As shown in Figure 4, the assimilated kinematics of the model by the proposed algorithm, and thus the resultant joint torques τ_*m*_ by the inverse dynamics were the exactly the same as those of the true kinematics and joint torques ${\tilde{\tau }}_{m}$ for *m* = {l/r-H,l/r-K,l/r-A}, while those by the naive algorithm exhibit substantial errors from the true kinematics and joint torques.

More specifically, Figures 4A–D show, respectively, the assimilated joint angles of the left leg for $\theta_{\text{HAT}}^{\text{(G)}}$, θ_l-H_, θ_l-K_, and θ_l-A_ by the naive and the proposed algorithms. In each panel, seven colored curves represent $\theta_{\text{HAT}}^{\text{(G)}}$ or θ_*m*_ that were estimated by the naive algorithm from each of the STA profiles for seven subjects shown in Figure 3, for which the colors in each panel of Figures 4A–D correspond to those in each panel of Figure 3. The black curves in Figures 4A–D, but visible only in Figures 4A,D, are those estimated by the proposed algorithm, and they were exactly the same with the true kinematics. In Figures 4B,C, the back curves for the proposed algorithm and the colored joint angles θ_l-H_ and θ_l-K_ for the naive algorithm are closely overlapped, meaning that the hip and knee joint angles are less-influenced by STA, regardless of the assimilation algorithms.

The left ankle joint angles (Figure 4D) estimated by the naive algorithm were largely different from the corresponding true joint kinematics, in comparison with the angles of the other joints (Figures 4B,C). It is noteworthy that the ankle joint angles estimated by the naive algorithm tended to be smaller than the true joint angles. Such large deviation from the true joint ankle angle was especially large for the subject 7 (red curve), although the STA Fourier coefficients for the subject 7 were not particularly deviated from those for the other subjects, except the cosine terms of *y*-element of marker-2 on the left-shank link (Figure 3F, red diamonds). Moreover, interestingly, despite the fact that the STA Fourier coefficients of the subject 4 for the cosine terms of *y*-element of marker-1 on the left-thigh link (Figure 3D green triangle) was particularly large, the assimilated kinematics for the subject 4 (Figure 4, green curves) were not particularly deviated from those for the other subjects. These observations imply non-trivial (nonlinear) relationships between the amounts of STA and the errors in the assimilated joint kinematics.

Figures 4E–G are joint torques of the left-hip (τ_l-H_), the left-knee (τ_l-K_), and the left-ankle (τ_l-A_), and Figures 4H–J show differences between the estimated joint torques (τ_*m*_) and the true joint torques ${\tilde{\tau }}_{\text{l-H}}$, ${\tilde{\tau }}_{\text{l-K}}$, and ${\tilde{\tau }}_{\text{l-A}}$ as the functions of time. Since the assimilated kinematics by the proposed algorithm was exactly the same with the true kinematics, the estimated joint torques were also exactly the same with the true joint torques, leading to the zero differences between them (the black horizontal lines in Figures 4H–J).

The joint torques estimated by the naive algorithm exhibited unignorable errors during stance phase, which can be confirmed in Figures 4H–J, where the gray shaded region in each panel indicates the stance phase of the left foot of the left leg. In all of seven STA profiles, the ankle joint angles and torques estimated by the naive algorithm showed similar error profiles and took smaller values than the true joint angles and torques. Such similarity between Figures 4D,G implies that the errors in the joint torques have its origin in the misestimation of the joint angles of the left ankle (Figure 4G), which propagates to the joint torques at the knee (Figure 4I) and the hip (Figure 4H), even though the estimated posture of HAT link and the hip and knee joint angles did not include large errors (Figures 4A–C). In case of the subject 7 (red curve), joint torques at the left-ankle (Figure 4G) took negative values during most of the left-leg stance phase, while the true joint torque of the left-ankle (the black curve in Figure 4G) took positive values for more than half of the stance phase. This error in the naive algorithm for the inverse dynamics analysis was absent in the proposed algorithm that removes the effect of STA.

## 5. Summary and discussion

### 5.1. Summary

In this paper, we proposed a simple but efficient algorithm to assimilate data of motion-captured marker positions affected by soft tissue artifact (STA) into models of multi-rigid-body systems. In the proposed algorithm, an unknown STA profile for each marker was Fourier-expanded, and its Fourier coefficients were determined so that the captured motion was optimally represented by a model of the rigid multi-link system. The determination of the Fourier coefficients of the STA profiles, i.e., the estimation of the non-measurable STA, allows to estimate the temporal changes in the global positions of the markers that are firmly fixed to the skeletal links (the landmark markers in this paper), leading to the STA-free assimilated motion that is consistent with the multi-rigid-link model. The key idea to determine the unknown STA was simply the periodicity assumption for the STA and kinematic constraints requiring that any two adjacent rigid-links are connected by a rotary joint.

To assess the efficiency of the proposed algorithm, we conducted a numerical experiment using a rigid seven-link model of human gait in the sagittal plane. In the experiment, we estimated kinematics of the model from seven sets of STA-affected marker positions, and showed that the proposed algorithm could determine the true kinematics of the model accurately for all sets of the STA profiles.

### 5.2. Difference between the proposed algorithm and similar algorithms

Since the proposed algorithm estimate kinematics of multi-rigid-body model using the joint constraints for the whole body, the proposed algorithm can be considered as a kind of the “global optimization”(Leardini et al., 2005) including multi-body kinematics optimization (Duprey et al., 2010; Richard et al., 2017) and kinematics estimation based on Kalman filter (Cerveri et al., 2003, 2005; Bonnet et al., 2017a). Here, let us clarify the novel aspect of the proposed algorithm complementary to the existing global optimization methods. In most studies of the global optimization, multi-rigid link models with virtual markers that are firmly fixed to the skeletal links are used. Kinematics of the multi-rigid-body system is optimized such that total distance between positions of captured markers and the corresponding virtual markers is minimized, under so-called “hard constraint” such as the joint constraint (Richard et al., 2017) or a physical constraint based on kinetic data (Cerveri et al., 2003, 2005). Since the cost function is the total distance between the captured and the virtual markers (Cerveri et al., 2003, 2005; Duprey et al., 2010), kinematics of the model is estimated such that motions of the virtual markers are similar to that of the corresponding captured markers, the STA of which are not treated in this process. Consequently, the estimated kinematics obtained by the global optimization can be strongly affected by the STA. That is, in this process, the larger STA amplitude, the stronger the effect on the kinematics estimation is. In contrast to these algorithms of the global optimization, in the proposed algorithm, an unknown STA profile is determined, and STA-free marker positions and the corresponding kinematics of a multi-rigid link model are estimated. This process is independent of the amplitude of STA, and can be used as long as the STA is periodic.

The major difference between the proposed algorithm and the other algorithms of the global optimization is whether STA profiles are explicitly determined or not. Since STA profiles are explicitly determined in the proposed algorithm, it may be claimed that we should use database of STA (Cereatti et al., 2017). Nevertheless, we believe that the proposed algorithm has some advantages over the algorithms that use the databases. Since STA is task-dependent and not reproducible for different subjects, effect of STA remains in kinematics estimated by the databases, unless a captured trial exactly matches the data in the database. In contrast, when we use the proposed algorithm, STA profiles are determined for each trial from the captured data, therefore, we do not need worry about task-dependent or subject-dependent STA, as long as the STA is periodic.

The proposed algorithm and the existing global optimization methods should be used complementary and collaboratively with other, rather than compared in their performance. Although we showed that the proposed algorithm for removing the STA can improve the naive algorithm as a very primitive global optimization method in this paper, a data assimilation can be performed better in general, if the proposed algorithm is utilized jointly with more sophisticated global optimization methods such as used in AnyBody (Andersen et al., 2009, 2010; Lund et al., 2015), among others.

### 5.3. Spurious solutions

Although the proposed algorithm succeeded to find the true kinematics of the human gait model for all examined STA profiles in the numerical experiment, it could fail depending on some other STA profiles. Since the minimization of the cost function $\tilde{F}$ of Equation (28) is a linear optimization problem, possible failures might be caused by the quadratic simultaneous equations arising from Equations (24–27) for solving the distance between two landmarks and the difference between the Fourier coefficients of STA. This is because the quadratic simultaneous equations have multiple solutions including the true and spurious solutions, and the spurious ones may be obtained if an initial condition for the solution search is not appropriate.

A promising way to set a good initial condition for solving the quadratic simultaneous equation is to use the naive algorithm based on the marker-coordinate system defined by Equations (6) and (7), which provide a rough estimation of the STA profiles without assuming the periodicity of the STA. We then expand the rough estimation of the STA into Fourier series, and utilize the obtained coefficients as the initial values of the STA Fourier coefficients for the proposed algorithm. Moreover, it is expected that the proposed algorithm would become more robust by finding appropriate positions of the landmarks for reducing a risk of being trapped by spurious solutions.

### 5.4. Extension to three-dimensional motions

An obvious issue that should be addressed in the future is to extend the proposed algorithm to three-dimensional motions and models. It would be able to achieve, because every processing performed in the proposed algorithm is independent of the dimensionality, but application of the algorithm to three-dimensional motions and models would just increase the number of markers (from two markers to three for each link) and unknown Fourier coefficients. That is, to specify a posture of a single rigid body in the three-dimensional space, at least three markers (marker-*ij* for *j* = {1,2,3}) should be attached on landmarks of the link-*i* (the landmark-*ij*) for a motion capture experiment.

However, there might be difficulty again in solving the quadratic simultaneous equations arising from Equations (24–27), particularly in the three-dimensional space. This is because the number of unknown parameters to be solved becomes larger than the number of the quadratic simultaneous equations if we consider distances between two markers on each link as in the two-dimensional case, leading to an ill-posed set of the quadratic simultaneous equations. Nevertheless, we still might be able to derive additional quadratic equations about distances between other pairs of markers among three markers on each link. Thus, it is expected that we can assimilate motion-captured kinematics of each link in the three-dimensional space into three-dimensional multi-link models using the proposed algorithm extended to the three-dimensional space.

## Author contributions

YS and TN designed the study, and wrote manuscript. YS and TI developed the proposed algorithm and the various computer programs.

### Conflict of interest statement

The authors declare that the research was conducted in the absence of any commercial or financial relationships that could be construed as a potential conflict of interest.

## Footnotes

^1^The origin and axes of the link-coordinate system can be defined in different ways. For example, the origin can also be at the center of mass (CoM) of the link-*i*, and the $x_{i}^{\left(\text{L}\right)}$-axis can be directed from the origin to distal end of the link as in Inoue et al. (2016).

## Appendix a

### Inverse dynamics analysis of rigid seven-link model

Motion equation of rigid seven-link model in the sagittal plane is described as follows

(A1) $$\begin{array}{r} \text{M}\left(\text{θ}\right)\ddot{\text{θ}}+\text{B}\left(\text{θ},\dot{\text{θ}}\right)+\text{K}\left(\text{θ}\right)=\text{F}+\tau , \end{array}$$

where **θ** is the vector of position and postures of all segments, **M** is the inertia matrix, **B** is the centrifugal and Coriolis force, **K** is the gravitational force and torque, **F** is the ground reaction force, and τ is the joint torque vector. See Yamasaki et al. (2003) or Fu et al. (2014) for details of elements of each matrix and vectors.

In the numerical experiment, we performed forward dynamics simulation, and obtained marker position data and ground reaction force. Subsequently, model kinematics (**θ**) were estimated by using old algorithm (“naive algorithm”) or new algorithm proposed in this study. From the estimated kinematics, joint angular velocities ($\dot{\theta }$) and joint angular accelerations ($\ddot{\theta }$) were calculated, and then, inertia matrix (**M**), vector of centrifugal and Coriolis force (**B**), and vector of gravitational force and torque (**K**) at each time instance were calculated. Finally, joint torques (τ) were calculated according to Equation (1).
